# A Multifunctional Peptide From *Bacillus* Fermented Soybean for Effective Inhibition of SARS-CoV-2 S1 Receptor Binding Domain and Modulation of Toll Like Receptor 4: A Molecular Docking Study

**DOI:** 10.3389/fmolb.2021.636647

**Published:** 2021-03-31

**Authors:** Srichandan Padhi, Samurailatpam Sanjukta, Rounak Chourasia, Rajendra K. Labala, Sudhir P. Singh, Amit K. Rai

**Affiliations:** ^1^Institute of Bioresources and Sustainable Development, Regional Centre, Gangtok, India; ^2^Institute of Bioresources and Sustainable Development, Imphal, India; ^3^Centre of Innovative and Applied Bioprocessing, Mohali, India

**Keywords:** fermented soybean, *Bacillus* spp., antiviral peptides, SARS-CoV-2, TLR4/MD2 complex, immunomodulation

## Abstract

Fermented soybean products are traditionally consumed and popular in many Asian countries and the northeastern part of India. To search for potential agents for the interruption of the Severe Acute Respiratory Syndrome Coronavirus 2 (SARS-CoV-2) Spike glycoprotein 1 (S1) and human angiotensin-converting enzyme 2 (ACE2) receptor interactions, the *in silico* antiviral prospective of peptides identified from the proteome of *kinema* was investigated. Soybean was fermented using *Bacillus licheniformis* KN1G, *Bacillus amyloliquefaciens* KN2G and two different strains of *Bacillus subtilis* (KN2B and KN2M). The peptides were screened *in silico* for possible antiviral activity using two different web servers (AVPpred and meta-iAVP), and binding interactions of selected 44 peptides were further explored against the receptor-binding domain (RBD) of the S1 protein (PDB ID: 6M0J) by molecular docking using ZDOCK. The results showed that a peptide ALPEEVIQHTFNLKSQ (P13) belonging to *B. licheniformis* KN1G fermented *kinema* was able to make contacts with the binding motif of RBD by blocking specific residues designated as critical (GLN493, ASN501) in the binding of human angiotensin-converting enzyme 2 (ACE2) cell receptor. The selected peptide was also observed to have a significant affinity towards human toll like receptor 4 (TLR4)/Myeloid Differentiation factor 2 (MD2) (PDB ID: 3FXI) complex known for its essential role in cytokine storm. The energy properties of the docked complexes were analyzed through the Generalized Born model and Solvent Accessibility method (MM/GBSA) using HawkDock server. The results showed peptidyl amino acids GLU5, GLN8, PHE11, and LEU13 contributed most to P13-RBD binding. Similarly, ARG90, PHE121, LEU61, PHE126, and ILE94 were appeared to be significant in P13-TLR4/MD2 complex. The findings of the study suggest that the peptides from fermented soy prepared using *B. licheniformis* KN1G have better potential to be used as antiviral agents. The specific peptide ALPEEVIQHTFNLKSQ could be synthesized and used in combination with experimental studies to validate its effect on SARS-CoV-2-hACE2 interaction and modulation of TLR4 activity. Subsequently, the protein hydrolysate comprising these peptides could be used as prophylaxis against viral diseases, including COVID-19.

## Introduction

Coronavirus disease (COVID-19), a pneumonia type outbreak which was first identified in Wuhan city of China, continues to devastate lives and livelihoods across the globe. The disease has been declared as Public Health Emergency on international concern by the WHO, and till date there have been 111, 762, 965 confirmed cases and 2, 479, 678 deaths globally^1^. The increasing prevalence of this disease has also been witnessed in India, accounting for about 11, 046, 914 identified cases including 1, 56, 705 deaths and recovery of 10, 738, 501 individuals^2^. The disease is caused by a novel transmissible human β-coronavirus known as Severe Acute Respiratory Syndrome Coronavirus 2 (SARS-CoV-2). Through its homo-trimeric spike glycoprotein (S1 and S2 subunit in each spike monomer) on the envelope, the virus enters the human cells and binds to host ACE2 cell receptors. Extensive analyses have unveiled the binding of SARS-CoV-2 and ACE2 is mediated by receptor-binding domain (RBD) on the surface of S1 glycoprotein, which is a fundamental step for the virus entry (Lan et al., 2020; Shang et al., 2020; Tai et al., 2020). Therefore, blocking the RBD or manipulating its essential residues crucial in binding hACE2 could ascertain potential therapeutics to prevent SARS-CoV-2 entry and its further infection.

The current shreds of evidence suggest that cytokine storm in SARS-CoV-2 infected patients as a result of over-activation of TLR could be an important factor in disease progression, even leading to multiple organ failure and death (Brandão et al., 2020; Huang et al., 2020; Sun et al., 2020). Toll like receptor 4 (TLR4), one of the extensively researched receptors which recognize a wide range of substances such as lipopolysaccharide (LPS) from various antigens, including viruses, is believed to cause intensive cytokine storm upon its interaction with the SARS-CoV-2 spike protein (Choudhury and Mukherjee, 2020). Myeloid Differentiation factor 2 (MD2), a co-receptor of TLR4, governs the activation of TLR4 during the infection. It is required to sense most of the LPS lipid chains. Therefore, inhibitors targeting the TLR4/MD2 complex could limit the over-activation of TLR4 and regulate the cytokine storm induced by SARS-CoV-2.

Fermented foods consumed in different parts of the globe have gained attention due to bioactive compounds formed on hydrolysis or transformation (Sanjukta and Rai, 2016; Rai et al., 2017). The Sikkim Himalayan region has a massive hold of traditional fermented foods owing to its prosperous ethnic diversity and rich bioresources (Das et al., 2016). *Kinema* is a non-salted sticky and naturally fermented alkaline soybean food, rich in protein including essential amino acids and is widely consumed among the ethnic people of Sikkim (Moktan et al., 2008). The constituent proteins are broken down by the action of microbial proteolytic machinery during the fermentation process to release small peptides that are known as food-derived peptides. These peptides could also be generated either by enzymatic hydrolysis or food processing. Over the decades, they have been established to be associated with several essential metabolic effects *in vitro* and *in vivo* (Rai et al., 2017; Chourasia et al., 2020a). These peptides are reported to have antioxidant, antihypertensive, antidiabetic, anticancer, and immunomodulatory properties (Chatterjee et al., 2018). Furthermore, many such peptides have been evolved as effective and selective viral entry inhibitors of deadly viruses like influenza (She et al., 2008; Albericio and Kruger, 2012), Herpes Simplex Virus (HSV) (Jenssen, 2005; Jaishankar et al., 2015), hepatitis (Abe et al., 2007), and Human Immunodeficiency Virus (HIV) (Pessi et al., 2009; Lee et al., 2014). Moreover, *in silico* based screening and analyses of natural or synthetic molecules against disease-linked protein targets are becoming increasingly popular nowadays and, in many cases, lead to the identification of potential drug candidates (Bruno et al., 2019). The molecular docking studies have extensively been used to efficiently predict the intermolecular interactions between a target protein and various ligands of interest and mimic stability of these structured complexes in a physiological environment (Olgac et al., 2017; Theerawatanasirikul et al., 2020).

In the present study, the peptides identified from soybeans fermented using *Bacillus* spp., namely *Bacillus licheniformis* KN1G, *Bacillus amyloliquefaciens* KN2G and two different *Bacillus subtilis* (KN2B and KN2M) were screened for their *in silico* antiviral activity. The selected 44 sequences were explored for their binding affinity towards critical residues on the SARS-CoV-2 RBD and TLR4/MD2 proteins using blind molecular docking. The novelty of this work lies in using and investigating peptides present in fermented soy, possibly interrupting SARS-CoV-2 RBD-hACE2 receptor interaction and inhibiting TLR4 activation. These peptides have the potential for anti-SARS-CoV-2 therapeutics development.

## Materials and Methods

### Sample Details

Yellow soybeans were fermented using proteolytic strains of *Bacillus* spp., including *B. subtilis* KN2M, *B. subtilis* KN2B, *B. licheniformis* KN1G, and *B. amyloliquefaciens* KN2G using protocol described earlier by Rai et al. (2017). Briefly, soybean seeds were soaked in distilled water overnight, cleaned and dehulled manually. Soaked seeds were cooked and transferred to an Erlenmeyer flask (250 mL) and sterilized in an autoclave. Starters for fermentation of the soybean seeds were prepared by inoculating the flasks containing autoclaved soybeans (20 gm) with actively growing cultures. The flasks were incubated at 42°C for 24 h and transferred to respective containers containing 200 gm of cooked soybeans, mixed properly and incubated 42°C for 24 h for fermentation. After the incubation, the seeds were oven-dried at 60°C and ground to powder for extract preparation. Lyophilized aqueous extracts of fermented soybean powder were used for the proteomics analysis using liquid chromatography-mass spectrometry (LC-MS/MS).

### Receptor Details

The S1-RBD of the SARS-CoV-2 was used as one of the receptors in the present study. The SARS CoV-2 RBD constituted a twisted five strand anti-parallel β-sheets (β_1_, β_2_, β_3_, β_4_, and β_7_) connecting themselves by short helices and loops, forming the core region (Lan et al., 2020). The 3D structure of RBD having 193 amino acids numbered as THR333… GLY526, was prepared from the RBD-human ACE2 receptor complex (PDB ID: 6M0J; 2.45 Å) retrieved from RCSB Protein Data Bank (PDB). More specifically, a structure motif (SER438… GLN506) called receptor binding motif (RBM) within the RBD is eventually responsible for the direct interaction with hACE2 receptor. Extensive sequence and structural analyses performed on the receptor usage of RBD have disclosed several critical residues that are known to play an essential role in ACE2 receptor recognition and cell entry; these include LEU455, PHE486, GLN493, SER494, and ASN501. Besides, the infectivity of SARS-CoV-2 was synchronized by the presence of two hot spots on RBD–ACE2 interface (31 and 353), stabilized by the amino acid residues LEU455, GLN493, and ASN501. In particular, GLN493 is the principal residue in recognizing the hACE2 receptor and infecting human cells, which is largely favorable for RBD-hACE2 interaction (Shang et al., 2020; Wan et al., 2020). Herein, the above-mentioned residues were considered and targeted for the inhibition of SARS-CoV-2 S1 RBD.

The crystal structure of the extracellular domain (ECD) of human TLR4 in complex with MD2 was used as the other receptor for docking. The ECD is a horseshoe-shaped structure consisting 603 amino acids comprising a concave and a convex surface. The concave surface consists of parallel β-sheets, while the loops and 3_10_ helices collectively make up the convex surface (Park et al., 2009). MD2 consists of 142 amino acids arranged in a β cup fold structure and comprised of two anti-parallel β sheets forming a hydrophobic pocket where LPS binds (Kim et al., 2007). The amino acid residues in the MD2 pocket such as ARG90, TYR102, SER120, LYS122, GLY123, PHE126, LYS130, and CYS133 were reported to play an essential role in mediating its binding with TLR4 (Gradisar et al., 2007; Peluso et al., 2010; Roh et al., 2011; Wang et al., 2015). Specifically, F126 located at the TLR4/MD2 interface, is the crucial residue initiating the dimerization and activation of TLR4 (Park et al., 2009). Therefore, blocking of these residues in the MD2 pocket is targeted in the present study.

### *In silico* Antiviral Activity Prediction and Selection of Peptides

The peptides identified from LC-MS/MS analyses were screened for their *in silico* antiviral activity using two different web servers AVPpred (Thakur et al., 2012) and meta-iAVP (Schaduangrat et al., 2019). AVPpred relies on a peptides dataset, experimentally checked for antiviral activity targeting important human viruses like influenza, HIV, Hepatitis C virus (HCV), and SARS, etc. It uses a model that have exploited various peptides sequence features, i.e., motifs and alignment, followed by amino acid composition (AAC) and physicochemical properties during five-fold cross-validation, using support vector machine (SVM) models. At the same time, meta-iAVP uses “effective feature presentation” that was extracted from a set of prediction scores derived from various machine learning algorithms and types of features such as AAC, pseudo amino acid composition (PseAAC), amphiphilic pseudo amino acid composition (Am-PseAAC), dipeptide composition (DPC), and g-gap dipeptide composition (GDC). The peptide sequences that were predicted to be antiviral by both the web tools were further computationally analyzed for physicochemical features, including toxicity, using PepCalc (Lear and Cobb, 2016) and ToxinPred (Gupta et al., 2013) web servers. All the computed features were used for further analyses.

### Molecular Docking

The molecular docking experiments were carried out as per the protocol suggested by Chourasia et al. (2020b). Three-dimensional structures of the selected peptide sequences were predicted using PEPstrMOD (Singh et al., 2015) and PEPFOLD (Thévenet et al., 2012). Similarly, the 3D structure of RBD was prepared from the RBD-ACE2 complex. Both the receptor (RBD) and peptide structures were optimized following the removal of water molecules and heteroatoms, the addition of missing residues and loops. This was done using the “prepare proteins” and “prepare ligands” protocol of Discovery Studio (DS) Client v20.1.019295. Molecular docking was performed using DS ZDOCK, a program that has been preferred and recommended suitable for studying protein-peptide interactions (Pierce et al., 2014; Chourasia et al., 2020b; Sanjukta et al., 2021). Cluster analysis was performed with an angular step size 15, an RMSD cutoff 6.0 and an interface cutoff 9.0. The maximum number of clusters and poses were, respectively set to 60 and 1000. The top poses of the largest cluster were further refined to obtain the low energy complex using DS RDOCK (Li et al., 2003), where a dielectric constant of 4.0 was used in the energy calculation. This procedure was repeated for every peptide. The final RBD-peptide complexes were examined for binding interactions between the peptidyl residues and critical amino acids on the receptor molecule. They were considered critical in the binding of RBD with hACE2. The selected peptides which found affinity towards any of the RBD critical residues were then investigated for their binding interaction against TLR4/MD2 structure complex. The crystal structure was optimized, and docking was performed, as mentioned above. Computation of energy parameters for selected complexes was carried out by employing the Generalized Born model and Solvent Accessibility method (MM/GBSA) using HawkDock server (Weng et al., 2019).

## Results

### *In silico* Antiviral Activity Prediction and Selection of Peptides

**FIGURE 1:**
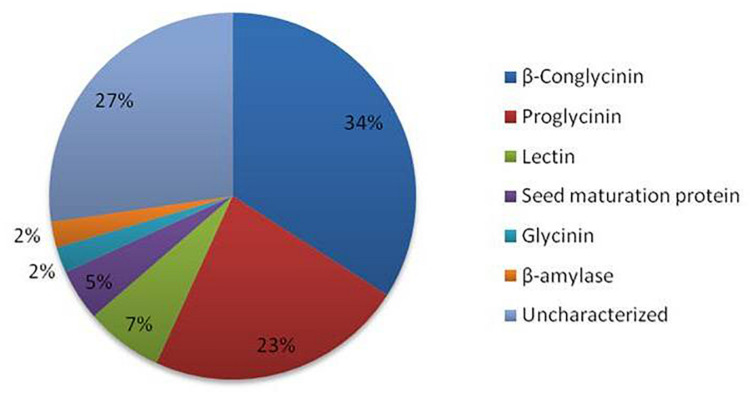
Antiviral peptides identified in fermented soybean and their source proteins.

The LC-MS/MS analyses of *kinema* produced using *B. licheniformis* KN1G, *B. amyloliquefaciens* KN2G and two different *B. subtilis* (KN2B and KN2M) strains resulted in identification of 3328 peptide sequences. *In silico* antiviral activity of these peptides, using two different web servers, showed 44 peptides to be antiviral with a probability 1.00. The majority of them derived from β-conglycinin and proglycinin (Figure 1). Furthermore, *kinema* produced *B. licheniformis* KN1G had a higher content of antiviral peptides (2.15% of total peptides), followed *B. subtilis* KN2B (0.76%), *B. subtilis* 2M (0.62%), and *B. amyloliquefaciens* KN2G (0.40%). The amino acid sequence and computed physicochemical properties of all 44 peptides described in the present study are given in Table 1. The calculated molecular weights for the selected peptides ranged from 1079 to 3927. The peptides demonstrated varying values of theoretical pI with a minimum 4.14 to a maximum 10.0. Similarly, the calculated Grand Average Hydropathy (GRAVY) of all except two peptides was positive. Sequence-based toxicity prediction using ToxinPred showed that the peptides from fermented soybean were non-toxic.

**TABLE 1:** Peptide sequences, source hydrolysate and computed physicochemical features.

Peptide Sequence(s)	Mol. Wt.	GRAVY	Toxicity
***B. licheniformis* KN1G fermented soybean**			
TSLDFPALSWLRL (P1)	1518.78	0.538	NO
SWTEWAKEKLSEGL (P2)	1663.85	−0.957	NO
TSLDFPALWLLKLS (P3)	1603.92	0.814	NO
KLGKFFEITPE (P4)	1308.54	−0.327	NO
KFVPKQPNMIL (P5)	1314.65	−0.073	NO
KFVPKQPNMILQ (P6)	1442.78	−0.358	NO
GGSQSQKGKQQE EENEGSNIL (P7)	2247.32	−1.833	NO
LPEGPAVKIGEN KDAMDGWFRLE (P8)	2572.92	−0.609	NO
NALKPDNRI ESEGGFIE (P9)	1889.05	−0.894	NO
LAFPAGSAQDIE NLIKNQRE (P10)	2214.46	−0.545	NO
LAFPGSAKDIENLI KSQSESYFVD (P11)	2658.94	−0.221	NO
AFPGSAKDIE NLIKSQSE (P12)	1934.13	−0.583	NO
ALPEEVIQH TFNLKSQ (P13)	1854.09	−0.425	NO
SWNKFVPK QPNMIL (P14)	1702.05	−0.429	NO
SLLNALPEEVIQHT FNLKSQQAR (P15)	2639.99	−0.422	NO
QEQEFLKYQ (P16)	1212.32	−1.789	NO
ANIELVGIKEQQQK QKQEEEPLE (P17)	2709.01	−1.439	NO
DIENLIKSQ (P18)	1079.18	−0.656	NO
VGIKEQQQKQQ KEEEPLE (P19)	2168.39	−2.011	NO
GNQEQEFLK (P20)	1092.17	−1.689	NO
FKLEFEPPFRIKSNQ (P21)	1880.18	−0.907	NO
LVGIKEQQQRQQ (P22)	1454.65	−1.442	NO
IPVNKPGRFE (P23)	1156.35	−0.750	NO
LAFPGSAKDIENLIKSQS ESYFVDAQPQQKEEGN (P24)	3769.09	−0.894	NO
ASYDTKFEEINKVLFS REEGQQQGEQRLQE (P25)	3587.86	−1.433	NO
DRPSIGNLAGANSLLNA LPEEVIQHTFNLKSQ (P26)	3447.85	−0.306	NO
STQAQQSYLQGFSH NILETSFHSEFEE (P27)	3145.30	−0.841	NO
SGFAPEFLKEAFGVNMQI VRNLQGENEEEDSGAIVT (P28)	3927.31	−0.306	NO
SGFTLEFLEHAFSVDKQ IAKNLQGENEGEDKGAIVT (P29)	3923.30	−0.428	NO
FLKEAFGVNMQIVRNL QGENEEEDSGAIVTVK (P30)	3566.00	−0.281	NO
EFLEHAFSVDKQIAKNLQ GENEGEDKGAIVTVK (P31)	3645.04	−0.6	NO
AFPGSAQAVEKLLKNQ RESYFVDAQPKKKEEGN (P32)	3708.15	−1.124	NO
MQGGKKAGESIKETA ANIGASAKAGME (P33)	2635.99	−0.511	NO
***B. amyloliquefaciens* KN2G fermented soybean**			
SLEDEISWFK (P34)	1253.37	−0.580	NO
FEEINKVLFGR (P35)	1351.57	−0.109	NO
***B. subtilis* KN2B fermented soybean**			
ISSEDKPFNLR (P36)	1305.45	−1	NO
NIVETFEENLGGIGEK (P37)	1748.91	−0.438	NO
LAGNQEQEFLK (P38)	1276.41	−0.873	NO
VIVELSKEQIR (P39)	1313.56	0.136	NO
GNQEQEFLK (P40)	1092.17	−1.689	NO
***B. subtilis* KN2M fermented soybean**			
SAKGKKGAFKGLNVA VKVIPKAKMTTA (P41)	2744.38	−0.044	NO
SLEDEISWFK (P42)	1253.37	−0.580	NO
GNQEQEFLK (P43)	1092.17	−1.689	NO
KPSAPKIPLE (P44)	1079.30	−0.680	NO

*GRAVY–grand average of hydropathy; Mol. Wt.–molecular weight in Dalton.*

### Peptide-RBD Docking

**FIGURE 2:**
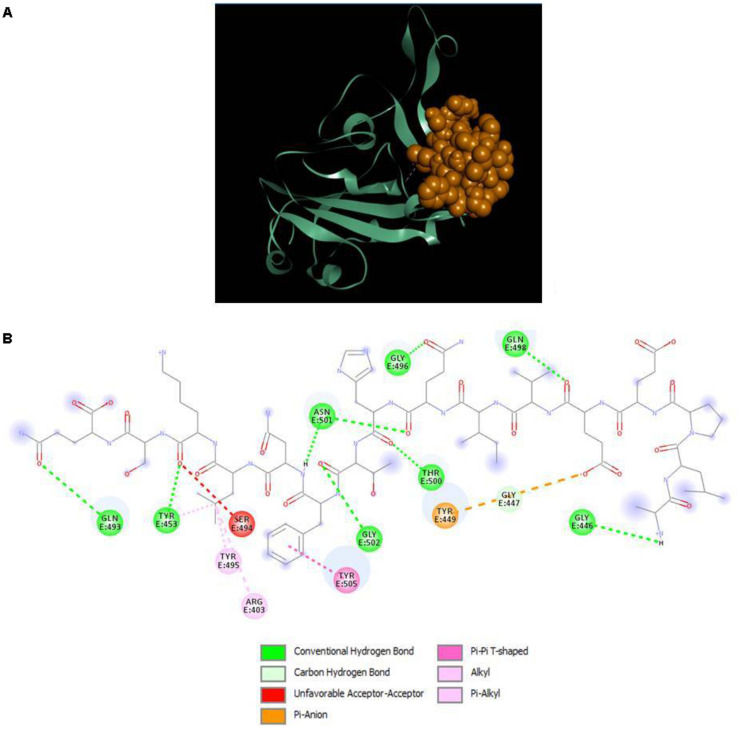
**(A)** Docking of RBD (ribbon) and peptide P13 (CPK), **(B)** 2D representation of RBD-peptide interactions. GLN493 and ASN501 are designated as critical to RBD-hACE2 binding.

**FIGURE 3:**
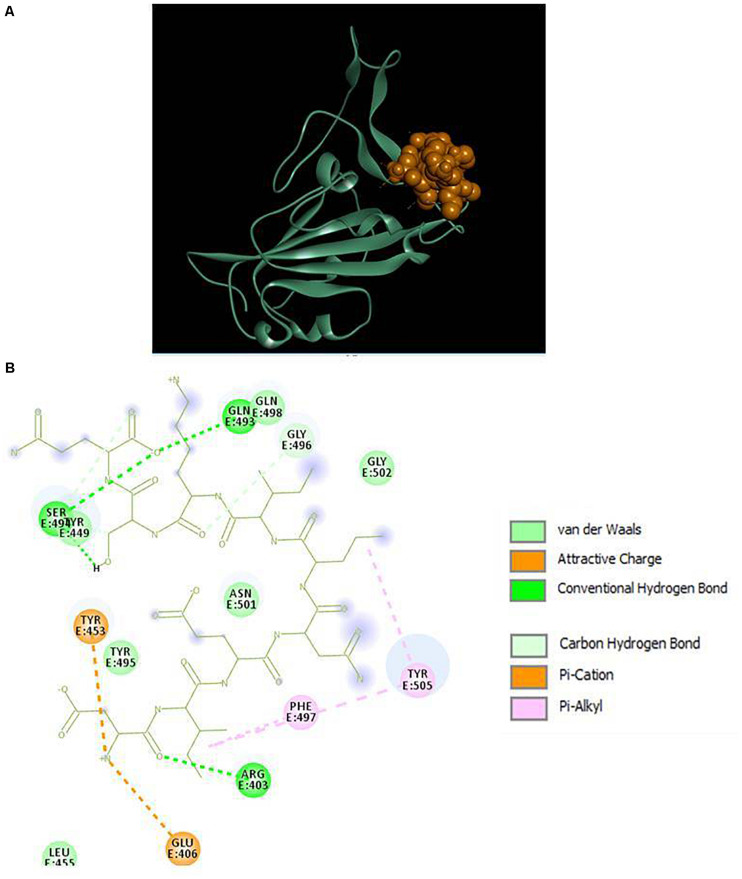
**(A)** Docking of RBD (ribbon) and peptide P18 (CPK), **(B)** 2D representation of RBD-peptide interactions. GLN493 and SER494 are designated as critical to RBD-hACE2 binding.

The results displayed amino acid sequences of 27 peptides made intermolecular contact with the receptor-binding motif on the RBD surface. Furthermore, two peptides designated as P13 (ALPEEVIQHTFNLKSQ) and P18 (DIENLIKSQ) belonging to *B. licheniformis* KN1G fermented soybean was able to interact with the targeted critical residues. The predicted RDOCK energies of the P13-RBD and P18-RBD docked complexes, respectively, were −9.30099 and −4.07086 kcal/mol. Similarly, the MM/GBSA energies of P13 and P18 bound RBD complexes were −48.03 and −35.83 kcal/mol, respectively. The peptide P13 was wholly bound to the RBM residues by virtue of nine conventional hydrogen bonds and six other interactions, including pi-pi, alkyl, pi-alkyl, and a covalent carbon-hydrogen bond (Figure 2). Among 10 (out of 16) peptidyl residues that participated in binding with the RBM, three were observed to be bonded to the critical residues GLN493, ASN501 with three conventional hydrogen bonds. The binding of P18 with RBM resulted into 11 non-bonded interactions (four conventional hydrogen, three carbon-hydrogen, four pi-linked and an attractive charge), linking seven of its amino acids to five RBM residues (Figure 3). The binding of P13 with the RBD was substantial as compared to P18 where GLU5, GLN8, PHE11, and LEU13 contributed the most negative free energy to the complex. The non-bonded interactions are detailed in Table 2.

**TABLE 2:** Details of non-bonded interactions between the peptides and SARS-CoV-2 receptor binding domain.

Peptide-Receptor Binding Domain (RBD) non-bonded interactions							

Peptide P13: ALPEEVIQHTFNLKSQ (ZDOCK Score: 11.18, E_RDOCK: −9.30099 kcal/mol, E_MM/GBSA: −48.03 kcal/mol)				Peptide P18: DIENLIKSQ (ZDOCK Score: 9.62, E_RDOCK: −4.07086 kcal/mol, E_MM/GBSA: −35.83 kcal/mol)			
	
Peptidyl	RBD	Types	Distance (Å)	Peptidyl	RBD	Types	Distance(Å)
GLN16	GLN493	Conventional hydrogen	2.80	GLN9	GLN493	Conventional hydrogen	2.38
LYS14	TYR453	Conventional hydrogen	1.97	GLN9	SER494	Conventional hydrogen	2.82
LEU13	TYR453	Pi-Alkyl	5.45	GLN9	SER494	Carbon hydrogen	3.51
LEU13	ARG403	Alkyl	5.06	SER8	SER494	Conventional hydrogen	2.87
LEU13	TYR495	Pi-Alkyl	5.47	SER8	SER494	Carbon hydrogen	3.01
PHE11	TYR505	Pi-Pi T shaped	4.38	LYS7	GLY496	Carbon hydrogen	3.45
THR10	GLY502	Conventional hydrogen	2.82	LEU5	TYR505	Pi-Alkyl	4.13
ASN12	ASN501	Conventional hydrogen	2.72	ILE2	TYR505	Pi-Alkyl	5.50
GLN8	ASN501	Conventional hydrogen	2.78	ILE2	PHE497	Pi-Alkyl	5.35
HIS9	THR500	Conventional hydrogen	1.88	ASP1	TYR453	Pi-Cation	4.22
GLN8	GLY496	Conventional hydrogen	2.49	ASP1	ARG403	Conventional hydrogen	2.03
GLU5	GLN498	Conventional hydrogen	2.04	ASP1	GLU406	Attractive charge	4.21
ALA1	GLY446	Conventional hydrogen	2.48				
GLU5	TYR449	Pi-Anion	4.08				
GLU5	GLY447	Carbon hydrogen	3.13				

*E_RDOCK–Energy of the structure complex computed using RDOCK program.*

*E_MM/GBSA–Energy of the structure complex computed using Generalized Born model and Solvent Accessibility method.*

### Peptide-TLR4/MD2 Docking

**FIGURE 4:**
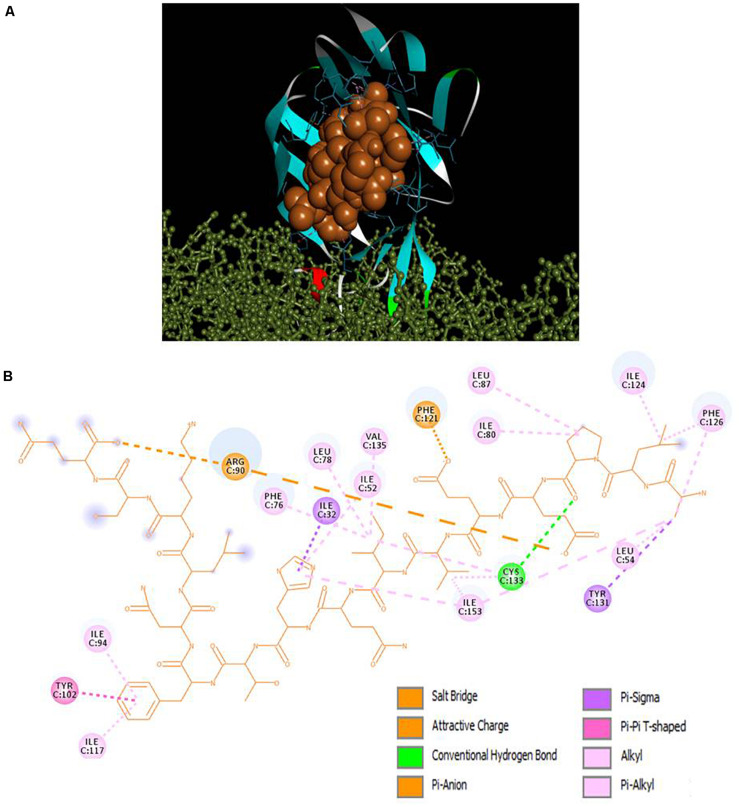
**(A)** Docking of TLR4 (ball and stick)/MD2 (ribbon) complex with peptide P13 (CPK), **(B)** 2D representation of TLR4/MD2-peptide interactions. ARG90, TYR102, PHE126, and CYS133 are designated as critical to TLR4/MD2-LPS binding.

**FIGURE 5:**
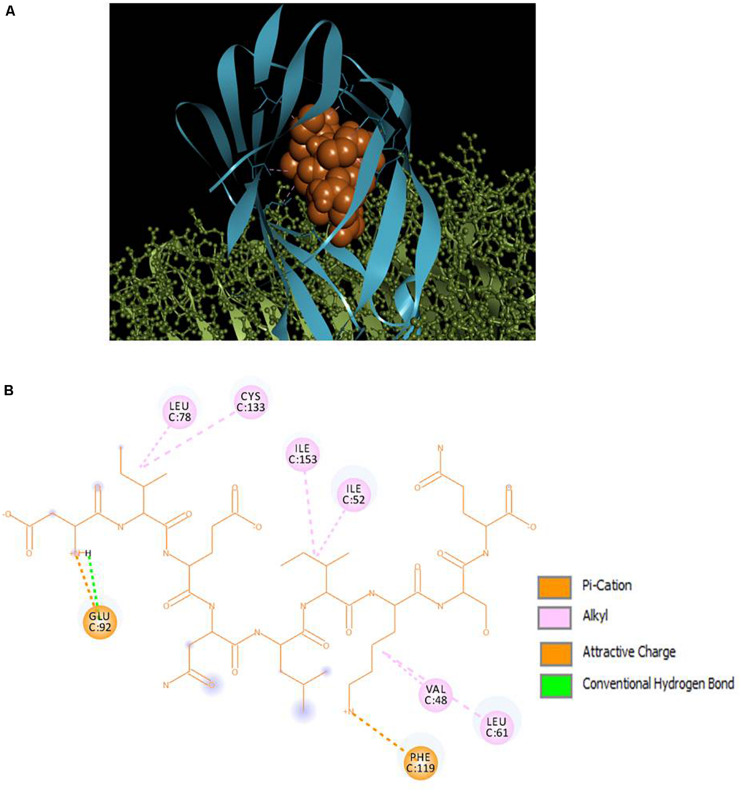
**(A)** Docking of TLR4 (ball and stick)/MD2 (ribbon) complex with peptide P18 (CPK), **(B)** 2D representation of TLR4/MD2-peptide interactions. CYS133 is designated as critical to TLR4/MD2-LPS binding.

**FIGURE 6:**
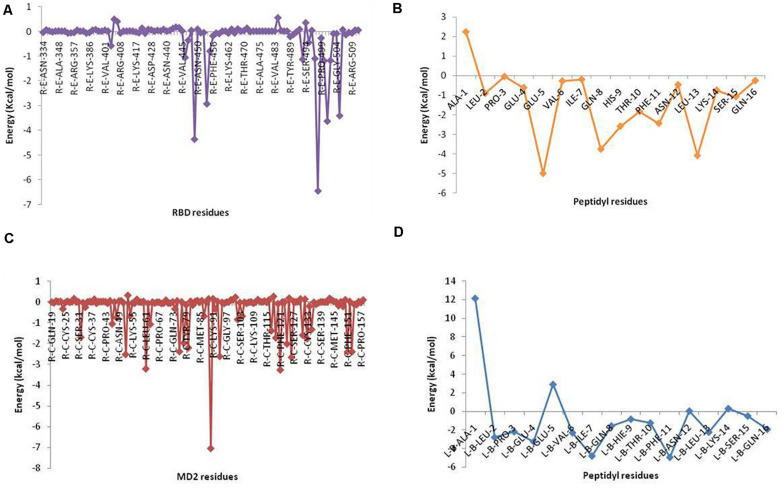
Binding free energy contribution of **(A)** RBD residues and **(B)** P13 residues in P13-RBD complex, and **(C)** MD2 residues and **(D)** P13 residues in P13-TLR4/MD2 complex. Energies were computed from MM/GBSA analysis.

Selected peptides were also examined for their possible affinity against TLR4/MD2 complex. P13 exhibited 18 interactions (Figure 4), while P18 bonded inside the cavity with the support of nine interactions with the residues inside the MD2 hydrophobic cavity (Figure 5). P13 was found to be bonded with ARG90, TYR102, PHE126, and CYS133. These residues are considered critical in TLR4/MD2 binding; however, P18 showed binding to CYS133 and others inside the pocket. The predicted RDOCK energies for P13-TLR4/MD2 and P18-TLR4/MD2 complexes were −23.464 and −9.76314, respectively. Similarly, the MM/GBSA energies of P13 and P18 bound TLR4/MD2 complexes were −59.66 and −40.97 kcal/mol, respectively. The detail of receptor-ligand interactions has been given in Table 3. Among the two peptides, receptor-ligand interactions involving P13-TLR4/MD2 complex were significant, and ARG90, PHE121, LEU61, PHE126, and ILE94 contributed to the binding free energy of the complex. The free binding energies contributed by the selected peptide P13, and the receptors in P13-RBD and P13-TLR4/MD2 complexes are given in Figure 6.

**TABLE 3:** Details of non-bonded interactions between the peptides and TLR4/MD2 complex.

Peptide-TLR4/MD2 complex non-bonded interactions							

Peptide P13: ALPEEVIQHTFNLKSQ (ZDOCK Score:13.12, E_RDOCK: −23.464 kcal/mol, E_MM/GBSA: −59.66 kcal/mol)				Peptide P18: DIENLIKSQ (ZDOCK Score:10.44, E_RDOCK: −9.76314 kcal/mol, E_MM/GBSA: −40.97 kcal/mol)			
	
Peptidyl	TLR4/MD2	Types	Distance (Å)	Peptidyl	TLR4/MD2	Types	Distance (Å)
PHE11	ILE94	Pi-Alkyl	4.48	ASP1	GLU92	Conventional hydrogen	1.89
PHE11	TYR102	Pi-Pi T shaped	5.70	ASP1	GLU92	Charge-Charge	4.90
PHE11	ILE117	Pi-Alkyl	4.98	ILE2	LEU78	Alkyl	4.36
GLN16	ARG90	Salt bridge	1.86	ILE2	CYS133	Alkyl	5.17
GLU4	ARG90	Salt bridge	4.27	ILE6	ILE52	Alkyl	5.36
ILE7	PHE76	Pi-Alkyl	5.27	ILE6	ILE153	Alkyl	4.73
HIS9	ILE32	Pi-Sigma	3.90	LYS7	PHE119	Pi-Cation	4.12
HIS9	ILE52	Pi-Alkyl	4.27	LYS7	LEU61	Alkyl	4.99
ILE7	LEU78	Alkyl	9.95	LYS7	VAL48	Alkyl	4.62
ILE7	VAL135	Alkyl	4.11				
GLU5	PHE121	Pi-Anion	3.48				
HIS9	ILE153	Pi-Alkyl	4.84				
ALA1	ILE153	Alkyl	4.28				
VAL6	CYS133	Alkyl	4.04				
ILE7	CYS133	Alkyl	5.00				
PRO3	CYS133	Conventional hydrogen	3.63				
ALA1	TYR131	Pi-Sigma	3.82				
ALA1	LEU54	Alkyl	4.14				
ALA1	PHE126	Alkyl	4.59				
LEU2	ILE124	Alkyl	4.67				
PRO3	LEU87	Alkyl	4.85				
PRO3	ILE80	Alkyl	4.14				

*E_RDOCK–Energy of the structure complex computed using RDOCK program.*

*E_MM/GBSA–Energy of the structure complex computed using Generalized Born model and Solvent Accessibility method.*

## Discussion

The COVID19 disease outbreak caused by SARS-CoV-2 stands for a pandemic threat to public health globally. Since this outbreak, millions of people have been infected, and many have lost their lives. Additionally, over activation of systemic immunity due to SARS-CoV-2 infection results in the cytokine storm, especially striking in severely ill patients. Further, millions of lives and livelihoods have been affected as a consequence of mandatory isolation and quarantine measures. In this way, the disease has brought significant challenges to the global health systems and economy. The prospects for the treatment of this infection lie in discovering specific agents (antivirals) that could target the inhibition of the virus and regulate the cytokine storm in the host (immunomodulators). Therefore, researchers worldwide are racing to find a possible intervention using functional foods and natural compounds as pharmaceuticals. In view of this, scores of studies have investigated the possible antiviral effects of several natural metabolites, including organic compounds and peptides, and drugs against SARS-CoV-2 protein targets (Luo et al., 2020; Vincent et al., 2020; Padhi et al., 2021; Yu et al., 2021).

Fermented soy products have been recommended as a possible therapy for SARS-CoV-2 infected patients in Japan. The recent data shows a decrease in death rates with daily intake of these products (Bayindir and Bayindir, 2020). Besides, several bioactive peptides, isolated and identified from fermented soy products, have been reported to exhibit antiviral activities against HIV, HSV, influenza virus, and human respiratory illness virus (Yamai et al., 2003; Wei et al., 2015; Peng et al., 2019). Some of these peptides have also been evaluated as effective immunomodulators (Yimit et al., 2012; Kiewiet et al., 2018). The fermentation process using dissimilar starter cultures could produce peptides of variable AAC and variable physical and chemical properties; however, bioactive aspects of these peptides need to be elucidated. In this study, the food peptides originated from the fermented soybean using different species and strains of *Bacillus* (*B. licheniformis* KN1G, *B. amyloliquefaciens* KN2G and two different *B. subtilis* KN2B and KN2M) were screened against the experimental antiviral peptide datasets for the prediction of their antiviral activity. The selected peptides were further evaluated for their binding interaction with the RBD of SARS-CoV-2 S1 glycoprotein, a conserved viral protein known to be significant in binding with ACE2 receptor and cell infection.

The strains used in the study were isolated from traditionally fermented *kinema* based on amylase, β-glucosidase and protease producing ability (Rai et al., 2017). Amylase and β-glucosidase are considered critical for fermentative property and enhancement of free polyphenols, respectively. Proteases were considered as specific hydrolysis of protein can lead to peptides in *kinema* with desired health benefits as a different starter can lead to the production of different types of peptides (Sanjukta and Rai, 2016). Therefore, isolates capable of producing *kinema* with controlled hydrolysis of protein without affecting the flavor and taste were selected. Sequence-based prediction of antiviral activity resulted in 44 peptides to be antiviral with a high probability. Several studies have reported the antiviral activity of fermented soybean fractions associated with oligopeptides released as a result of microbial proteolysis of soy proteins (Andres et al., 2009; Singh et al., 2017). Most of the predicted antiviral peptides were originated from β-conglycinin and proglycinin (precursor protein of glycinin). The results are supported by the fact that glycinin and β-conglycinin largely contribute to fermented soybean’s bioactive peptides (Tsuruki et al., 2003; Matemu et al., 2011). These peptides are the hydrolyzed products of soy proteins, and their production relies on the proteolytic ability of a specific starter (Verni et al., 2019). In our study, *kinema* produced using *B. licheniformis* KN1G produced the highest number of antiviral peptides suggesting the strain capable of producing peptides of antiviral significance during fermentation.

Hydrophilicity is often associated with the solubility of protein or peptides as a good solubility level prevents the peptide from aggregation, and allows the peptide to stay intact and promotes its interaction with the microbial lipid membrane (Miron et al., 2019). In this investigation, 41 out of 44 peptides were hydrophilic (as indicated by their negative GRAVY index), signifying their aqueous solubility is considerable to interact with the viral membranes. Besides, amphipathicity also determines ability of an antiviral peptide to disrupt the lipid membranes and interact with their hydrophilic-hydrophobic interface from its aqueous phase (Edwards et al., 2016). Few peptides described in this study were amphipathic having GRAVY value between −0.5 and +0.5, which could be considered as a potential ligand in penetrating the viral membrane. GRAVY index is also an indicator of hemolytic activity or cell toxicity. There are experimental evidences on highly hydrophobic peptides (GRAVY value > 1) causing hemolysis *in vitro*, and the same with low hydrophobicity were not reported to cause any toxic effects even at high concentrations (Yin et al., 2012). In this study, three peptides were predicted to be hydrophobic and having GRAVY index within 0.9, displaying low toxicity. Furthermore, toxicity of the peptides was validated *in silico*, and all were predicted to be non-toxic.

*In silico* docking studies have been proven to be extremely useful in facilitating the structural diversity of natural metabolites, including oligopeptides, to be exploited in a controlled manner (Pintilie and Stefaniu, 2018). DS ZDOCK program has been used for docking, an initial-stage docking program for protein-protein complexes but can also be used for protein-peptide docking. This algorithm searches for orientational space by rotating the ligand around its geometric center with keeping the receptor fixed in space (Chen et al., 2003; Kufareva et al., 2011; Ning et al., 2018; Baruah and Bose, 2020). All the 44 selected peptides were docked on the surface of SARS-CoV-2 receptor binding domain for selecting peptides with the best possible type of interactions and binding affinities with the targeted critical residues. Two peptide fragments ALPEEVIQHTFNLKSQ and DIENLIKSQ designated as P13 and P18, were shown to interact with the RBM following several interactions (covalent and non-covalent). The selected peptides were also docked against the TLR4/MD2 complex, where both of them were found to fit into the hydrophobic cavity of MD2 with the support of various non-covalent contacts.

Non-covalent forces play an essential role in pharmaceutical drug designing and lead optimization (Rahman et al., 2016). Apart from the conventional hydrogen bonds, those are believed to have a potential impact on the ligand’s binding affinity. Pi-sigma interactions (Pi-alkyl, Pi-pi, and Pi-anion) aid in charge transfer and help intercalate the ligand on the receptor binding site (Arthur and Uzairu, 2019). The strength of these forces is substantial in the P13-RBD and P13-TLR4-MD2 complexes, supported by low binding energies, including RDOCK and MM/GBSA energies. DS RDOCK was developed for protein-protein complexes and has subsequently been used for predicting protein-peptide interactions (Devarapalli et al., 2012; Karhu et al., 2015; Prabhudesai et al., 2018). DS RDOCK is based on a force field CHARMM that operates limited molecular dynamics to fine-tune receptor-ligand complexes from ZDOCK. During such refinement, van der Waals energy is first calculated for discarding the docking poses with clashes. Then, scoring of the poses is done based on de-solvation and electrostatic energies (Karhu et al., 2015).

GLN493, an amino acid on the surface of RBD is reportedly the most critical element in mediating the interaction between the SARS-CoV-2 S1 RBD and hACE2. Blocking or inhibiting this residue could be an important strategy in interrupting the virus-host receptor interaction and preventing the virus from entering the host cell (Shang et al., 2020; Wan et al., 2020). The peptide P13 displayed interaction with GLN493 with a conventional hydrogen bond that is believed to confer stability and strength to the interaction, further ascertaining its potential to prevent the virus from accessing the receptor interface and cell entry.

Human TLR4/MD2 complex recognizes the LPS released by pathogenic microbes; and its interaction with LPS activates the TLR4 to release pro-inflammatory cytokines (Song et al., 2020). In many circumstances, over activation of TLR4 and overproduction of cytokines has been observed as detrimental to health and lead to multi-organ failure (Song et al., 2020; Tang et al., 2020). However, structural changes in the TLR4/MD2 complex could halt the binding of LPS in the hydrophobic cavity and modulate the immune response in the host system. Several studies have targeted this receptor to investigate cytokine production regulation *in vitro* and *in vivo* (Zhang et al., 2016, 2019; Thomas et al., 2020). The intermolecular interaction between our peptide (P13) with the critical amino acids (PHE126, CYS133, ARG90, and TYR102) of the MD2 hydrophobic pocket could affect the TLR4/MD2-LPS binding, thereby regulating TLR4 activation and cytokine production.

The current investigation portrays the computational prediction of fermented soy-derived peptides for antiviral properties and characterization of selected antiviral peptides as inhibitors of SARS-CoV-2 S1 RBD and human TLR4/MD2 complex. The peptide sequences can be chemically synthesized and linked with *in vitro* studies to evaluate their toxic effects and antiviral efficacy. Furthermore, the peptide that showed good binding affinity towards the SARS-CoV-2 S1RBD and TLR4/MD2 structure complex could be useful when combined with experimental cellular and animal models to get a complete understanding of its action mechanisms related to viral inhibition and modulation of host immune responses. The findings may further lead to the development of peptide-based therapeutics upon successful laboratory and clinical examinations to overcome the present pandemic. Also, the soybean fermented using the strain *B. licheniformis* KN1G that produced the most number of antiviral peptides, could be used as a prophylactic measure towards treating viral infections, including COVID-19.

## Conclusion

Soybean fermented using *Bacillus* spp. revealed production of specific antiviral peptides based on *in silico* analysis. Production of different antiviral peptides was dependent on the starter culture at species as well as strain level. Further analyses of the selected peptides using molecular docking studies demonstrated that two peptides could interact with the critical residues of SARS-CoV-2 S1 receptor binding domain and the human TLR4/MD2 complex. The findings could be used as the starting point to further investigate the *in vitro* and *in vivo* function leading to peptide-based anti-SARS-CoV-2 therapeutic development and immunomodulatory agents. Furthermore, the fermented soybean using *B. licheniformis* 1G with the highest number of antiviral peptides could be prophylactic against other viral infections. The present study opens an avenue for further exploring different microbial strains and protein-rich foods for the production of novel antiviral peptides for viral diseases including COVID19.

## Data Availability Statement

The original contributions presented in the study are included in the article/supplementary material, further inquiries can be directed to the corresponding author/s.

## Author Contributions

AR contributed to conception, design of the study, and finalization of manuscript. SP did a major part of proteomics and bioinformatics analysis and wrote the first draft of the manuscript. RC, RKL, and SS assisted in bioinformatics analysis and development of fermented products and wrote sections of the manuscript. SPS assisted in proteomics analysis and thoroughly reviewed the manuscript. All authors contributed to manuscript revision, read, and approved the submitted version.

## Conflict of Interest

The authors declare that the research was conducted in the absence of any commercial or financial relationships that could be construed as a potential conflict of interest.

## Footnotes

1 https://covid19.who.int

2 https://www.mohfw.gov.in
